# Molecular Landscape of Pediatric Low‐Grade Gliomas: Insights From RNA‐NGS and Bioinformatic Analysis

**DOI:** 10.1002/gcc.70085

**Published:** 2025-10-11

**Authors:** Petr Brož, Martina Strnadová, Denisa Olejníková, Johana Kotiš, Tereza Pospíšilíková, Adéla Mišove, Miroslav Koblížek, Josef Zámečník, Michal Zápotocký, David Sumerauer, Aleš Vícha, Martin Kynčl, Petr Libý, Vladimír Beneš, Lenka Krsková

**Affiliations:** ^1^ Department of Pediatric Hematology and Oncology 2nd Faculty of Medicine, Charles University and Motol University Hospital Prague Czech Republic; ^2^ Bioxsys Ltd Ústí nad Labem Czech Republic; ^3^ Department of Pathology and Molecular Medicine 2nd Faculty of Medicine, Charles University and Motol University Hospital Prague Czech Republic; ^4^ Department of Radiology 2nd Faculty of Medicine, Charles University and Motol University Hospital Prague Czech Republic; ^5^ Department of Neurosurgery 2nd Faculty of Medicine, Charles University and Motol University Hospital Prague Czech Republic; ^6^ Department of Genetics and Molecular Diagnostics Regional Hospital Liberec Liberec Czech Republic

**Keywords:** bioinformatics analysis, fusion genes, low‐grade glioma, RNA NGS

## Abstract

Pediatric low‐grade gliomas (pLGG) are the most common group of childhood brain tumors. Genetic alterations in the RAS–RAF–mitogen‐activated protein kinase (MAPK) pathway are the molecular drivers in the vast majority of pLGG. A large proportion of pediatric pLGG are characterized by the presence of fusion genes. An institutional molecular analysis together with an RNA‐NGS study was performed to reveal LGG‐associated molecular alterations. In our cohort of pLGG patients, molecular alterations were identified in 318 out of 342 cases (92.9%) through a combination of RT‐PCR, Sanger sequencing, and NGS methodologies. Fusion events were independently called using three fusion callers: Archer Analysis 6.0 and/or 7.0, Arriba version 2.4, and STAR‐Fusion 24. Among these, STAR‐Fusion had the lowest sensitivity, detecting rearrangements in only 67% of fusion‐positive cases. In contrast, Arriba detected rearrangements in 97.77% of cases, while Archer detected rearrangements in 88.6% of cases. These findings highlight differences in detection efficiency among fusion callers, emphasizing the importance of tool selection in molecular diagnostics. The detection of fusion genes is very important for correct diagnosis, prognosis, and adequate targeted treatment.

## 1 Introduction

Pediatric central nervous system (CNS) tumors are the most common (20%–25%) solid tumors in children. Within this group, pediatric low‐grade gliomas (pLGG) are the most common, accounting for more than 40% of all childhood CNS tumors [1]. They represent a heterogeneous group of tumors with different locations, ages at presentation, histologic subtypes, and clinical behavior.

Pediatric CNS tumors are mostly characterized by oncogenic fusions rather than multiple mutated genes and invariably involve activation of the mitogen‐activated protein kinase (MAPK) cascade [2, 3]. During development, the rat sarcoma‐virus family proteins/the mitogen‐activated protein kinase (RAS/MAPK) pathway are important in the formation of the cortex, midbrain, and cerebellum [4, 5, 6]. Its role in neurogenesis is particularly interesting with respect to glial pathogenesis, as the cell of origin for gliomas is now proposed to be a neural stem cell or neural progenitor rather than a post‐mitotic glial cell [6, 7, 8]. The B‐Raf protooncogene (*BRAF*) is the most commonly altered gene in pLGG with two predominant alterations: the *KIAA1549::BRAF* fusion and the *BRAF* V600E pathogenic variant, both resulting in activation of the *BRAF* kinase [1, 2, 3]. In addition to common pLGG alterations, rarer alterations affecting RAS/MAPK signaling were detected, including those involving *FGFR1–3*, *NTRK1–3*, *RAF1*, *ALK*, and *ROS1* [3, 4, 5, 6].

Only a small proportion of pLGG is characterized by some aberrations that indirectly affect the RAS/MAPK pathway, such as *MYB/MYBL1* alterations. MYB is involved in the control of the proliferation and differentiation of hematopoietic and other progenitor cells and is associated with proto‐oncogenic functions in both human leukemia and solid tumors [7]. MYBL1 is a member of the MYB family of proteins. *MYBL1* is not a known oncogene but is closely related to the proto‐oncogene *MYB* [8]. MYBL1 is involved in the positive regulation of transcription by RNA polymerase II.

Less commonly, alterations in Isocitrate dehydrogenase—*IDH1,2* are observed in pLGG [9]. The *IDH1* R132H mutation was found in only 0.8% of pLGG, with those identified occurring in older children/adolescents [10].

A large proportion of pediatric LGGs are characterized by the presence of fusion genes. Gene fusions are known to result in the formation of a hybrid protein that is either constitutively active or exhibits altered function. The most common example is the *KIAA1549::BRAF* fusion, first described in 2008 by Jones et al. [11]. The *KIAA1549::BRAF* fusion results in the tandem duplication of the *BRAF* gene, leading to the loss of the auto‐inhibitory domain of *BRAF* and the constitutive activation of its kinase domain [11, 12, 13]. The *KIAA1549::BRAF* fusion represents the molecular hallmark of pilocytic astrocytoma (PA). Since then, the omics revolution has enabled us to find many other driver‐gene transcript fusions with various fusion partners and genomic breakpoints, including but not limited to *BRAF*, *RAF1*, *NTRK1/2/3*, *FGFR 1/2/3*, *ROS1*, *EGFR*, *ALK*, and *PDGFRA* [14, 15].

More than half of the reported unique fusions are intrachromosomal (54%), such as the *KIAA1549::BRAF* fusion mentioned above, or large deletions, such as those causing the recurrent *FAM131B::BRAF* fusion. The duplication of the kinase domain of *FGFR1* (KDD *FGFR1*) may be another intrachromosomal rearrangement typical of LGG, consisting of a ~4.5 kb internal tandem duplication of the portion of the gene encoding the kinase domain [5]. However, due to the difficulties in detecting intrachromosomal fusions, their incidence number might be underrepresented and might therefore increase with improved detection algorithms [16].

Molecular analysis, particularly next‐generation sequencing (NGS), is essential to: confirm diagnosis, guide treatment, provide prognostic information, and enable patient recruitment to basket trials based on a tumor's molecular signature. Nevertheless, bioinformatic analysis of raw data obtained from NGS can be challenging and necessitates a range of approaches.

Therefore, an institutional molecular analysis together with an RNA‐NGS study was performed to reveal LGG‐associated molecular alterations and their therapeutic implications. Another goal was to test and compare different callers for the detection of fusion genes, kinase domain duplications, and splice variants.

## 2 Material and Methods

### 2.1 Patient Cohort

To determine the molecular alterations in pLGG, we analyzed 362 non‐NF1 tumors (tumors from children without known neurofibromatosis type 1 syndrome). Overall, 94.47% (*n* = 342/362) had sufficient material for molecular profiling (with positive amplification).

The present study's cohort of pLGG consisted of 362 non‐NF1 patients who were followed and treated at Motol University Hospital from 2000 to 2024. The median age at diagnosis was 8.16 years (range 0–20.2). Patients were followed on an outpatient or inpatient basis with regular MRI imaging. The institutional review board approved the study, and all patients gave informed consent according to our routine procedure.

### 2.2 Histopathology and Immunohistochemistry

The most representative FFPE block of tumor tissue was selected, and consecutive sections were immunohistochemically examined. The LGG cases were immunohistochemically studied using the following panel of primary antibodies: GFAP (1:1000, rabbit polyclonal, Dako, (Agilent Cat# N1506, RRID:AB_10013482)), NeuN (1:1000, mouse monoclonal, clone A60, Chemicon (Millipore Cat# MAB377, RRID:AB_2298772)), neurofilament protein (1:50, mouse monoclonal, clone 2F11, Dako, (Agilent Cat# M0762, RRID:AB_2314899)), CD34 (1:40, mouse monoclonal, clone QBEnd‐10, Dako, Agilent Cat# M716501‐2, RRID:AB_2750581), and Ki‐67 (1:150, mouse monoclonal, clone MIB‐1, Dako, Agilent Cat# M7240, RRID:AB_2142367). All immunohistochemical reactions were visualized by the PolyDetector DAB HRP Brown (BioSB) immunohistochemistry detection system.

### 2.3 Molecular Examination

**FIGURE 1:**
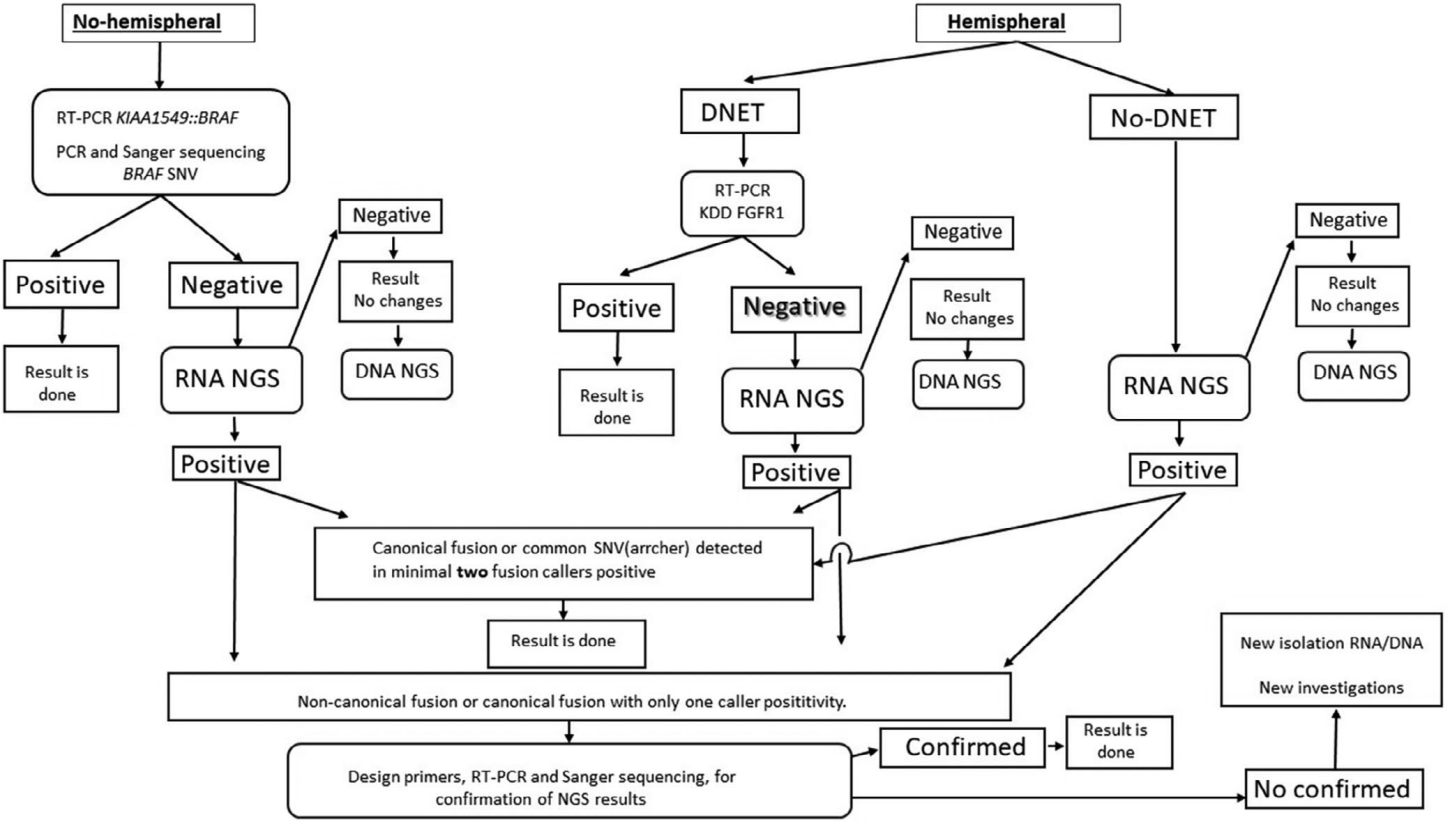
Scheme of a cost‐effective algorithm for the detection of molecular alterations in pLGG.

Tumor RNAs were purified from FFPE blocks using the High Pure FFPET RNA Isolation Kit (Roche Diagnostics) or from cryosections using Trizol (Invitrogen). To detect the most common alterations, we have used cost‐effective algorithms that include the initial use of RT‐PCR diagnostics or Sanger sequencing (Figure 1).

PCR and Sanger sequencing were conducted to examine hotspot mutations at codon 600 of *BRAF* ex15, codons 546 and 656 of *FGFR1* ex12, and of *FGFR1* ex14, respectively, using the previously described primer pairs [17, 18]. Amplification was performed using 2× PCRBIO HS Taq Mix Red (PCR Biosystems Ltd., London, UK). Direct Sanger sequencing was performed using BigDye Terminator v 3.1 chemistry (Life Technologies) and an ABI PRISM 3500Dx genetic analyzer (Applied Biosystems). The results were analyzed using Chromaslite 2.01 (Technelysium, Pty Ltd., Brisbane, Australia).

RT‐PCR was used to detect the expected *KIAA1549::BRAF* fusion because it was more cost‐effective and less time‐consuming. For the RT‐PCR, we used the primers published by Badiali et al. [19]. A similar approach was used to detect the V600E hot‐spot variant of the *BRAF* gene using mutation‐specific PCR and Sanger sequencing. This approach was used for posterior fossa, spinal cord, midline, and optic pathway tumors. For hemispheric LGGs, in most cases, we proceeded directly to diagnosis by NGS, as for the above localizations with negative findings by PCR techniques.

Molecular detection of the kinase domain duplication of *FGFR1* (KDD *FGFR1*) in the histologic diagnosis of DNET was also performed by RT‐PCR in the majority of cases [3].

In case of a negative result or hemispheric localization, we used NGS. FusionPlex Lung V1 (14 genes) or V2 (17 genes) panels, and in some cases the FusionPlex Pan Solid Tumor V2 (137 genes), Oncology Research (75 genes), and custom panel EMEA (57 genes) (ArcherDX) were used to prepare the NGS libraries. The final amplicons were subsequently sequenced on an MiSeq (Illumina) instrument. Archer FusionPlex also offers robust performance even for FFPE samples. RNA extraction, library preparation, and parallel sequencing were performed as per the manufacturer's recommendations. Fusion events were independently called using three fusion callers: Archer Analysis 6.0 and/or 7.0, Arriba, and STAR‐Fusion 24. Splicing Isoform was called using in‐house workflow. We then designed primers for the newly detected rearrangements and confirmed the new fusion genes by Sanger sequencing.

### 2.4 Bioinformatics Methodology

The bioinformatics analysis for pLGG was performed using a well‐established pipeline aimed at detecting gene fusions, kinase domain duplications, and alternative splicing events associated with tumorigenesis.

### 2.5 Reference Genome and Data Preparation

For the transcriptome analysis, we utilized the Gencode GTF file version 45 (GRCh38.p14) [20]. Raw sequencing data were first processed with Fastp, a fast and efficient tool for quality control and preprocessing of high‐throughput sequencing data [21]. To assess the global quality of the trimmed data, we used FastQC [22]. The complete bioinformatics pipeline is described in the Supporting Information.

### 2.6 Alignment and Fusion Detection

For sequence alignment, we used STAR (Spliced Transcripts Alignment to a Reference) version 2.7.9a, a widely used RNA‐seq aligner, to map the filtered FASTQ files to the GRCh38 reference genome. The alignment process was conducted with default parameters, and the junction file was generated inside the BAM file with hard clipping. One modification was made to the standard STAR settings by adjusting the –chimScoreDropMax parameter to 30, which refines the alignment for chimeric reads—those that map to multiple locations in the genome or have fusion‐gene signatures. This adjustment is intended to enhance the detection of gene fusions, a hallmark of pLGGs [23].

Fusion gene detection was performed using Arriba (version 2.4) [24] as well as STAR‐Fusion [24], both of which are popular tools for detecting fusion events in RNA‐seq data. Arriba was run with a modified parameter ‐E 0.8, which specifies the minimum expected number of supporting reads for a fusion event to be considered. This threshold ensures a higher specificity by minimizing the detection of false positives, particularly in cases where low‐abundance fusion transcripts are present. The use of a stringent threshold like 0.8 is critical in rare fusion detection, as it reduces noise from non‐specific alignments while maintaining sensitivity. Both fusion tools are known for their sensitivity in detecting clinically relevant fusions that drive tumorigenesis [16].

Additionally, we used STAR‐Fusion with default parameters for fusion gene detection. While both tools identify similar fusion events, the use of multiple fusion callers allows for cross‐validation and ensures the robustness of the results [25].

### 2.7 Detection of Splicing Events and Isoforms

In addition to gene fusions, we sought to identify smaller genetic events, such as exon skipping and alternative splicing patterns, which are often associated with cancer‐specific isoforms. These alterations are often difficult to detect with conventional exon‐based approaches but can be identified by analyzing chimeric junctions.

For this purpose, we developed an in‐house workflow designed to detect these splicing events by identifying chimeric junctions between exons, which represent the skipped regions of the genome.

## 3 Results

To determine the true frequency of molecular alterations in our pLGG cohort, we analyzed 342 tumors where material quality was sufficient for molecular analysis. In our group of LGG patients, we were able to detect molecular alterations in 318/342 patients (92.9%) using a combination of RT‐PCR, Sanger sequencing, and NGS techniques: *KIAA1549::BRAF* (*n* = 143/342, 41.81%), *BRAF* p.V600E (*n* = 59/342, 17.25%) together accounted for 59% of non‐NF1 pLGGs. Rare *BRAF* alterations, non‐canonical *BRAF* fusions (*n* = 14/342, 4.09%), and *BRAF* SNVs other than V600E (*n* = 7/342, 2%) accounted for an additional 6.14%. The next most common alterations were those affecting receptor tyrosine kinases—mainly *FGFR1–3*, including *FGFR1::TACC1* (*n* = 9), KDD *FGFR1* (*n* = 17), hot spot mutations in *FGFR1* (*n* = 13), *FGFR2* fusions (*n* = 12), and finally the rare *FGFR3::TACC3* fusion (*n* = 3). The *FGFR*‐altered LGG subgroup comprised 16.08% of the pLGGs studied. Other tyrosine kinase rearrangements like *ALK*, *ROS1*, *NTRK1*–*3*, and *RAF1* were rare (*n* = 18, 5.26%). SNVs in the *KRAS* gene were detected in four patients and a mutation in the *PDGFRA* gene in one patient. Non‐RAS/MAPK alterations such as *MYB* (*n* = 3) or *MYBL1* (*n* = 1) and *IDH1* SNVs (*n* = 11/342, 3.2%) were detected in a small proportion of our cohort.

### 3.1 *BRAF* Fusions

The most common *BRAF* fusion in pLGG was *KIAA1549::BRAF*. In our cohort, we detected nine different *KIAA1549::BRAF* exon‐exon junctions, including 16–9, 15–9, 16–11, 15–11, 13–11, 13–9, 10–9, 17–11, and 19–9, all resulting in the loss of the regulatory domain of the *BRAF* gene. The most common fusion was 16–9 (*n* = 71), the second was 15–9 (*n* = 43), and the third was 16–11 (*n* = 10). Other fusions were rare: 13–11 (*n* = 6), 15–11 (*n* = 6), 10–9 (*n* = 4), 13–9 (*n* = 1), 17–11 (*n* = 1), and finally 19–9 (*n* = 1). All 10–9 fusions were detected in spinal LGGs, and similarly, most *KIAA1549* exon 13 fusions were also detected in spinal LGG patients (*n* = 4). The next two *KIAA1549::BRAF* fusions, 13–11, were seen in cerebellar LGGs, and one in an optic pathway tumor. The majority of the typical 16–9 fusions was seen in the posterior fossa region, similar to the 16–11 fusion. *KIAA1549::BRAF*–positive tumors occurred in patients at a younger age (median age of 5 years), were predominantly located in the posterior fossa, and they were predominantly pilocytic astrocytoma histologically.

**FIGURE 2:**
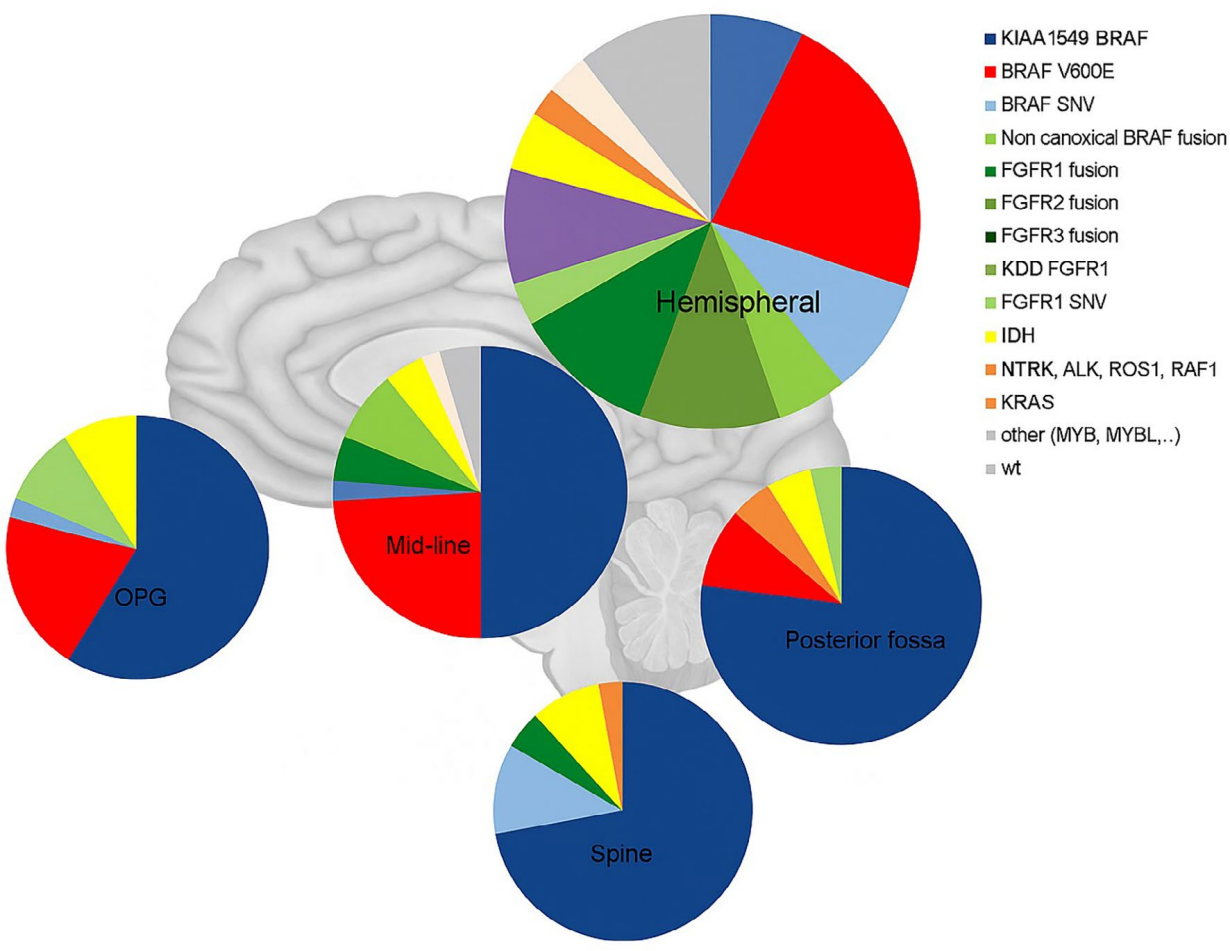
Pie charts showing the frequency of molecular alterations in pLGG without NF1 diagnosed between 2000 and 2024 according to tumor location.

We detected 13 different N‐terminal partners of the *BRAF* gene other than *KIAA1549* (*BCAS1*, *FAM131B*, *GNAI1*, *BCAS1*, *GTF21*, *MKRN1*, *NRF1*, *NUDCD3*, *PAG1*, *PRKAR2B*, *RNF130*, *TAX1BP1*, and a novel *EPHB2*). Most of the fusion breakpoints occur on exon 9 of *BRAF*, and, in all fusions, the inhibitory regulatory domain located in the first six exons is disrupted by the fusion. Half of the non‐canonical *BRAF* fusions were seen in hemispheric LGGs (Figure 2).

### 3.2 BRAF SNVs

The second most frequent alteration in the LGG group was the *BRAF* V600E mutation (*n* = 59/342, 17.25%). *BRAF* V600E tumors were more common in older patients (median age of 10.14 years) and were more likely to be located in hemispheric regions. However, in contrast to *KIAA1549::BRAF*, they were rare in the cerebellum, and they included a spectrum of histologic subtypes. SNVs in the *BRAF* gene other than V600E were rare. The mutation *BRAF* T599dup (*n* = 3) indicates the insertion of a duplicate amino acid, threonine, into the protein kinase of the BRAF protein. The next rare *BRAF* SNV was G469A, which is a hotspot mutation within the protein kinase domain of BRAF.

### 3.3 Other Direct Members of RAS/MAPK

A further 2.34% (*n* = 8) of cases contained alterations in other direct members of the RAS/MAPK pathway, including four *KRAS* SNVs and four *RAF1* fusions (*SRGAP3::RAF1*, *QKI::RAF1*, and *FCHCD2::RAF1*). *KRAS* is an upstream molecule in the RAS/MAPK pathway. Reports on the frequency of *KRAS* mutations in the pLGG range from 1%–5% and primarily arise in pilocytic astrocytoma [5, 26, 27, 28, 29]. Two of four patients with the *KRAS* mutation had hemispheric ganglioglioma, one had hemispheric PA, and the last one had spinal DLGNT (diffuse leptomeningeal glioneuronal tumor).

*RAF1* fusions contain an intact RAF1 kinase domain (encoded by exons 10–17) and are functionally similar to *BRAF* fusions [30]. Three out of four patients with a *RAF1* fusion had a PA, while one patient had a glioneuronal tumor.

### 3.4 FGFR1‐3 Alterations

*FGFR1‐3* are receptor tyrosine kinases (RTKs) that play a key role in signal transduction via activation of their intra‐membranous tyrosine kinase domain (TKD) [26]. *FGFR1* is the second most commonly altered gene in pLGG. *FGFR1* alterations in pLGG arise via three mechanisms: *FGFR1* mutations, *FGFR1::TACC1* fusions, and *FGFR1* kinase domain duplications [3, 5, 31, 32].

*FGFR1‐3* alterations were detected in 54 patients (16.8%), with a median age of 8.69 years. Among the *FGFR1* alterations, KDD *FGFR1*, which is typical for the diagnosis of DNET, was the most common (*n* = 17), with a median age of 6.78 years. The fusion gene *FGFR1::TACC1* was detected in nine patients, with a median age of 10.4 years. *FGFR1* hot spot mutations were observed in 13 patients (*FGFR1* K656E or D, N546 D or K) with a median age of 10 years. In most cases, one causal change is typical for pLGG, except for *FGFR1* SNPs, where we found either an additional *FGFR1* SNV or a combination with a *PIK3CA* gene SNV (E545K or H1047R) in 3 of 13 patients.

In our pLGG cohort, we observed a relatively high frequency of *FGFR2* fusion genes (*n* = 12), namely *FGFR2::INA* (*n* = 5), *FGFR2::KIAA1598* (*n* = 3), *FGFR2::ZCCHC24* (*n* = 2), *FGFR2::CTNNA3* (*n* = 1), and *FGFR2::PASD1* (*n* = 1). The median age of the patients with the *FGFR2* fusion was 6.84 years. All *FGFR2* rearranged patients had tumors located in the hemisphere (Figure 2).

In three patients, the *FGFR3::TACC3* fusion gene was detected, which is generally rare in the pLGG population. All were hemispherically located.

### 3.5 Other RTK Fusions

Fusions in other RTK were rare in pLGG (5.26%), with a median age of 5.45 years. *ALK*, *ROS1*, *NTRK*, and *MET* fusions are typical in infantile HGG [33] and are also found in a variety of pediatric gliomas [16]. *NTRK1*‐3 genes (*n* = 9) had the highest abundance among RTK fusions: *NACC::NTRK2*, *CLIP2::NTRK2*, *SPTAN1::NTRK2*, *KANK1::NTRK2*, *PDE4DIP::NTRK1*, *KIF21B::NTRK1*, *ETV6::NTRK3*, and finally *BCR::NTRK3*. *ALK* fusions included *ALK::GIGYF2*, *PPP1CB::ALK*, and *EML4::ALK*. All ALK‐positive pLGGs were located in the hemisphere, in contrast to the other RTK fusions, which were located in different CNS compartments. Paradoxically, all but one ALK‐fused LGG were not infantile LGGs. The other two *ALK* fusion‐positive patients were older than 10 years. *ROS1* fusions were represented by *GOPC1::ROS1* and *GIT2::ROS1*. The first case was a congenital hemispheric tumor and the second a midline tumor in a 3‐year‐old girl.

### 3.6 IDH SNVs

*IDH1* mutations are common in adult low‐grade gliomas. In pLGG, *IDH1* mutations were rare, accounting for only 3.2% of cases. The *IDH1* hot spot mutation was observed in 11 patients (R132H, R132C, R132G, R132S). Patients with *IDH* SNVs were diagnosed in late childhood (median age of 17.13 years), with the youngest patient diagnosed at 12.6 years. There was no difference in median age between *IDH1* variants.

### 3.7 RNA‐NGS Analysis

The rearrangement was identified in 88 of 115 pLGG cases analyzed by RNA NGS (74.1%). Fusion‐gene detection was performed using three bioinformatic tools: Archer, Arriba, and STAR‐Fusion. Among these, STAR‐Fusion demonstrated the lowest sensitivity, detecting rearrangements in only 59 of 88 fusion‐positive cases (67%). In contrast, Arriba identified 86 of 88 rearrangements (97.77%), while Archer detected 78 of 88 rearrangements (88.6%). These findings highlight differences in detection efficiency among fusion callers, emphasizing the importance of tool selection in molecular diagnostics (Table 1).

**TABLE 1:** Discordant results from three callers for the detection of fusion genes in the pediatric LGG population.

Dg.	Archer	Arriba	Star fusion	RT‐PCR
Astr. Gr2	KIAA1549::BRAF 16–11	KIAA1549::BRAF 16–11	X	KIAA1549::BRAF 16–11
PA	KIAA1549::BRAF 15–9	KIAA1549::BRAF 15–9	X	KIAA1549::BRAF 15–9
DA	KIAA1549::BRAF 15–9	KIAA1549::BRAF 15–9	X	KIAA1549::BRAF 15–9
PA	GNAI1::BRAF 1–10	GNAI1::BRAF 1–10	X	GNAI1::BRAF 1–10
PA	KIAA1549::BRAF 13–11	KIAA1549::BRAF 13–11	X	KIAA1549::BRAF 13–11
PA	X	KIAA1549::BRAF 15–9	X	KIAA1549::BRAF 15–9
PA	KIAA1549::BRAF 10–9	KIAA1549::BRAF 10–9	X	KIAA1549::BRAF 10–9
PA	KIAA1549::BRAF 16–9	KIAA1549::BRAF 16–9	X	KIAA1549::BRAF 16–9
Astr. Gr2	KIAA1549::BRAF 13–9	KIAA1549::BRAF 13–9	X	ND
GG	X	EPHB2::BRAF 9–10	EPHB2::BRAF	EPHB2::BRAF 9–10
PA	X	KIAA1549::BRAF 16–9	X	KIAA1549::BRAF 16–9
GG	X	NRF1::BRAF 10–10	X	ND
PA	KIAA1549::BRAF 15–9	X	X	KIAA1549::BRAF 15–9
PA	ETV6::NTRK3 5–15	ETV6::NTRK3 5–15	X	ND
PA	PDE4DIP::NTRK1 16–12	PDE4DIP::NTRK1 16–12	X	PDE4DIP::NTRK1 16–12
PA	NACC2::NTRK2 5–13	NACC2::NTRK2 5–13	X	NACC2::NTRK2 5–13
LGG NOS	X	PAG1::BRAF 5–9	PAG1::BRAF	PAG1::BRAF 5–9
GG	PPP1CB::ALK 5–20	PPP1CB::ALK 5–20	X	PPP1CB::ALK 5–20
GG	FGFR2::KIAA1598 17–7	FGFR2::KIAA1598 17–7	X	FGFR2::KIAA1598 17–7
PA	TAX1BP1::BRAF 5–9	TAX1BP1::BRAF 5–9	X	TAX1BP1::BRAF 5–9
DNET	X	truncat. MYBL1	X	ND
DNET	FGFR3::TACC3 17–10	FGFR3::TACC3 17–10	X	FGFR3::TACC3 17–10
GG	FGFR2::KIAA1598 17–7	FGFR2::KIAA1598 17–7	X	FGFR2::KIAA1598 17–7
DNET	FGFR2::ZCCHC24 17–2	FGFR2::ZCCHC24 17–2	X	FGFR2::ZCCHC24 17–2
PA	KIAA1549::BRAF 13–11	KIAA1549::BRAF 13–11	X	KIAA1549::BRAF 13–11
DNET	X	KDD FGFR1	X	KDD FGFR1
PA	X	KDD FGFR1	X	KDD FGFR1
PA	X	KDD FGFR1	X	KDD FGFR1
LGG	MKRN1::BRAF 4–11	X	X	MKRN1::BRAF 4–11
DNET	X	KDD FGFR1	X	KDD FGFR1
infant. glioma	GIT2::ROS1 16–36	GIT2::ROS1 16–36	X	GIT2::ROS1 16–36

The duplication of the kinase domain of the *FGFR1* gene (KDD *FGFR1*) proved to be the most challenging and could only be detected using the Arriba caller.

**FIGURE 3:**
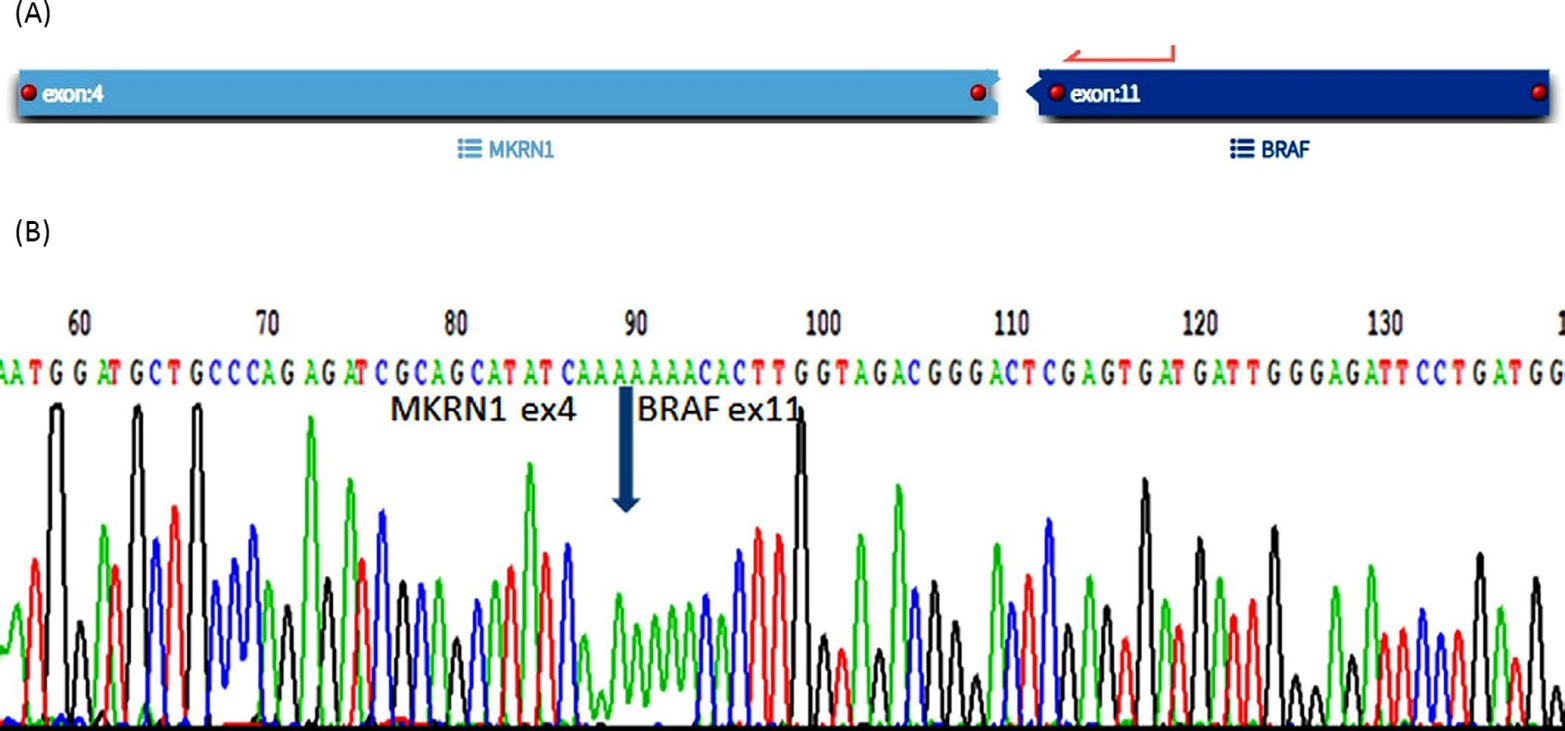
(A) Schematic visualization of the detected fusion transcript *MKRN1::BRA*F using the Archer software. (B) RT‐PCR and sequencing analysis of *MKRN1::BRAF* positive LGG.

In addition, the use of multiple callers was successful in detecting *BRAF* rearrangements, both *KIAA1549::BRAF* and non‐KIAA *BRAF* fusions. For the two LGGs with low‐quality RNA, Archer software failed to detect the *KIAA1549::BRAF* fusion, but the Arriba caller was successful. Similarly, for the rare non‐KIAA *BRAF* fusion genes, we were able to detect *EPHB2::BRAF* and *PAG1::BRAF* fusions using Arriba and the STAR‐Fusion caller. Another *NRF1::BRAF* fusion was again only detected with the Arriba caller. On the other hand, the *MKRN1::BRAF* fusion gene was not detected by either the Arriba caller or STAR‐Fusion, but it was detected by Archer. The result of Archer analysis was confirmed by RT‐PCR and subsequent Sanger sequencing (Figure 3).

**FIGURE 4:**
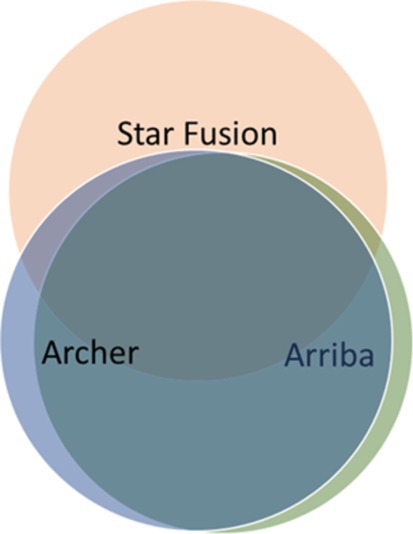
Venn diagram. The Venn diagram schematically summarizes the success rate of the individual callers used.

Archer FusionPlex kits work on the principle of amplicon NGS and can be successfully used on archived FFPE material of lower quality. We were able to demonstrate a *KIAA1549::BRAF* fusion with exon 16–9 in a patient with recurrent PA 33 years after diagnosis. Using Archer FusionPlex Lung, we were subsequently able to demonstrate the same fusion in 33‐year‐old archived FFPE material, but, due to a very low QC score, the fusion was only detected by the Arriba caller. In this case, it is again well documented that even in low‐quality material, a combination of callers is needed to give us a better chance of detecting a causal fusion gene. Truncation of several genes, e.g., a *MYBL1* truncating rearrangement, was also detected only by Arriba. In contrast, oncogenic variants of genes, such as *EGFR*vIII and *MET* exon 14 skipping, were more often missed by Arriba and STAR‐Fusion compared to Archer (data not shown). For the detection of these changes, it is advisable to use an additional caller for the detection of splice variants. The Venn diagram (Figure 4) schematically summarizes the success rate of the individual callers used.

**FIGURE 5:**
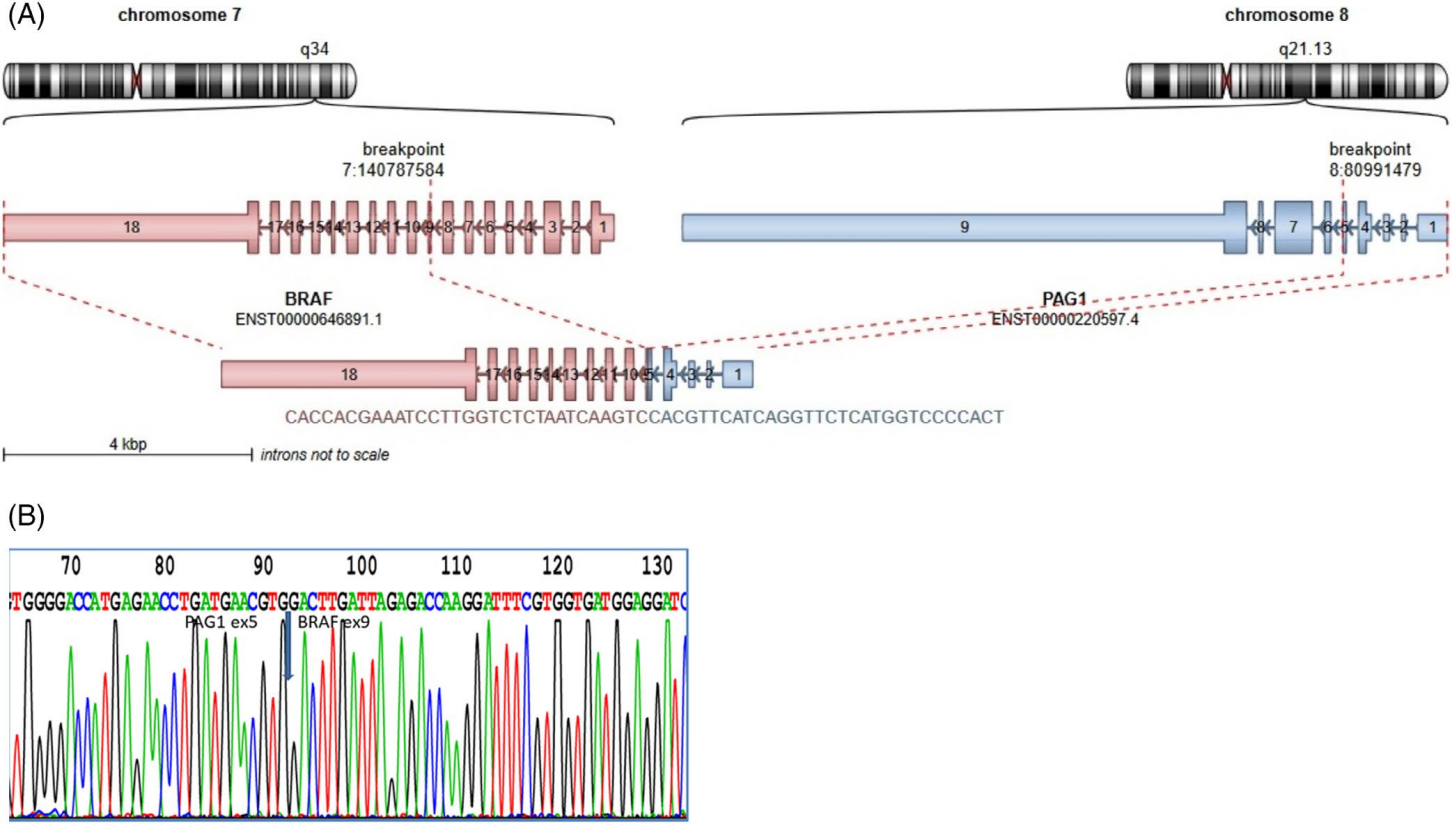
(A) Schematic visualization of *PAG1::BRAF* fusion transcript detection with Arriba software (https://github.com/suhrig/arriba/). (B) RT‐PCR and sequencing analysis of *PAG1::BRAF* positive LGG.

All discordant rearrangements were confirmed by RT‐PCR followed by Sanger sequencing. Similarly, novel or rare fusions were confirmed when we first designed primers. After RT‐PCR, the final product was verified by Sanger sequencing (Figure 5).

## 4 Discussion

With the recent 5th edition of the WHO classification for tumors of the CNS, which emphasizes the role of molecular diagnostics, the detection of fusion genes has become an important diagnostic marker in pediatric CNS neoplasms [34]. Based on our experience with LGG molecular diagnostics, we developed a cost‐effective algorithm that combines targeted testing (RT‐PCR or Sanger sequencing for the most common alterations) with NGS multicalling in negative cases (Figure 1).

Our findings confirm that the majority of genetic events in pLGG converge on the MAPK signaling pathway. The most prevalent were *KIAA1549::BRAF* fusions (41.8%), *BRAF* V600E mutations (17.25%), and *FGFR1* alterations (16.8%). RNA sequencing revealed nine distinct *KIAA1549::BRAF* exon–exon junctions as well as 15 rare or novel *BRAF* fusions, including intrachromosomal rearrangements on chromosome 7 (*NUCD3::BRAF*, *MKRN1::BRAF*, *FAM131B::BRAF*, *TAX1BP1::BRAF*, *PRKAR2B::BRAF*, *NRF1::BRAF*) and translocation‐derived fusions such as *RNF130::BRAF* [t(5;7)], *BCAS1::BRAF* [t(7;20)], *PAG1::BRAF* [t(7;8)], and *GTF21::BRAF* [t(7;7)] [13, 35, 36, 37, 38, 39, 40, 41, 42, 43, 44]. Notably, we report for the first time an *EPHB2::BRAF* fusion in a pediatric brain tumor. The *EPHB2* gene encodes a member of the Eph‐receptor family of receptor tyrosine kinases, which are composed of an N‐terminal ligand‐binding domain, a transmembrane region, and an intracellular kinase domain. Fusion with *EPHB2* has previously been described with other partners, such as *NTRK1* [45, 46], *PDZD4* [46] or *NBPF3* [47], *ASAP3* [45] or *INTS11* [48]. Similar to other *BRAF* fusions, the resulting protein EPHB2::BRAF lacks the N‐terminal inhibitory domain, leading to constitutive BRAF kinase activity and enhanced MEK/ERK signaling.

Detection of *BRAF* fusions required the combined use of multiple bioinformatic callers, as lower transcript expression frequently led to false negatives. Arriba proved most reliable, missing only 2 of 47 fusion‐positive cases compared with 4 missed by Archer and 14 by STAR‐Fusion. Failures occurred across both intrachromosomal and translocation‐derived fusions, including canonical *KIAA1549::BRAF*, underscoring the importance of a multicaller strategy. Even canonical fusions like *KIAA1549::BRAF* can be difficult to detect, especially in suboptimal samples. Determining an optimal cut‐off for fusion‐gene detection is challenging, especially when dealing with suboptimal FFPE samples with degraded RNA. The QC is determined by Archer Analysis, so that the number of unique RNA start sites for the GSP2 control must be greater than 10. In our cohort of pLGG patients, we detected the fusion genes by Archer Analysis, even in 10 cases where the sample failed QC (seven cases of *KIAA1549::BRAF*, 1 case of *GNAI1::BRAF*, 1 case of *NACC2::NTRK2*, and 1 case of *PPP1CB::ALK*). This fact clearly indicates the importance of using an additional caller, since in all cases, the fusion gene was detected at least by the Arriba caller and RT‐PCR.

Beyond fusions, we also detected rare *BRAF* SNVs. In addition to V600E, approximately half of which occurred in hemispheric tumors, we observed T599dup (*n* = 3), which enhances MEK/ERK phosphorylation, and G469A, a known hotspot mutation that activates the MAPK pathway independently of RAS [49, 50, 51, 52].

The second most common alterations in our cohort were *FGFR* fusions/SNVs, with an overall prevalence of *FGFR* alterations of 16.8%—notably higher than the 6.1% reported by Ryall et al. [4]. This may reflect the higher proportion of patients from the epilepsy program, as epilepsy‐associated tumors are enriched for *FGFR* gene alterations. *FGFR1* alterations occurred via three mechanisms: kinase domain duplication (KDD), oncogenic fusions with *TACC* partners, and hotspot mutations [3, 5, 31, 32]. *FGFR1* KDD, the most frequent, results in constitutive kinase activation [3, 53], while *FGFR::TACC* rearrangements also promote ligand‐independent activation via dimerization domains and have been reported across CNS tumors, including extraventricular neurocytoma, glioblastoma, IDH‐wildtype gliomas, and pLGG [3, 54, 55, 56]. In our cohort, we identified nine *FGFR1::TACC1* and three *FGFR3::TACC3* fusions, as well as 12 cases with *FGFR2* alterations, spanning polymorphous low‐grade neuroepithelial tumor of the young (PLNTY—7 cases), dysembryoplastic neuroepithelial tumor (DNT—2 cases), ganglioglioma (GG—2 cases), and multinodular and vacuolating tumor (MVNT, 1 case), confirming their heterogeneous histopathological spectrum and overall benign clinical behavior [28, 29, 57, 58]. We also detected five *FGFR1*‐mutant tumors, involving recurrent N546K and K656E variants, both of which increase kinase activity and confer oncogenic potential [59, 60, 61]. Prognostically, *FGFR1* KDD and *FGFR1/FGFR2* fusions are generally linked to favorable outcomes, whereas *FGFR1* mutations portend intermediate risk and inferior survival [53, 62]. From a technical standpoint, some *FGFR* alterations, particularly KDD events, are missed by individual callers, reinforcing the need for multicaller pipelines and splice‐aware algorithms. Clinically, their accurate identification remains essential both for diagnosis and for potential eligibility for FGFR‐targeted therapies.

Rare alterations in our cohort further expanded the genetic spectrum of LGG. We identified four *RAF1* fusions, including a novel *FCHSD2::RAF1*, in addition to *SRGAP3::RAF1* and *QKI::RAF1*. The breakpoint in *FCHSD2::RAF1* juxtaposed exon 8 of *RAF1* with exon 13 of *FCHSD2*, an adaptor protein involved in clathrin‐mediated endocytosis, resulting in constitutive RAF1 kinase activity. Although rare, *RAF1* fusions have been described in pilocytic astrocytoma and several extracranial tumors and consistently activate MAPK and PI3K pathways [63, 64, 65]. Their sensitivity to RAF and MEK inhibitors highlights their clinical significance.

**FIGURE 6:**
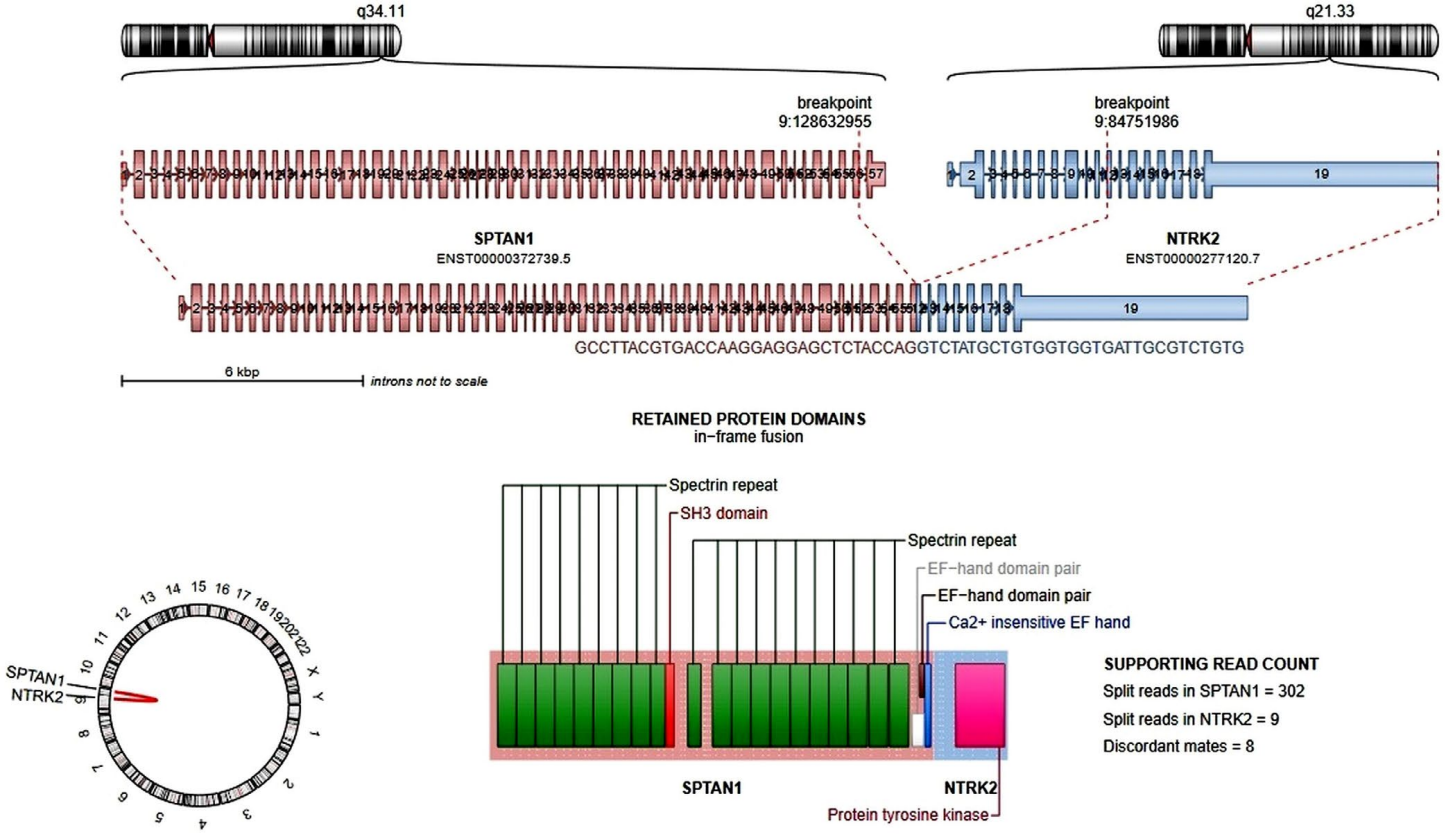
Schematic visualization using Arriba software (https://github.com/suhrig/arriba/) of the detected *SPTAN1::NTRK2* fusion transcript.

We also detected nine LGGs with *NTRK1–3* rearrangements, including two previously undescribed fusions: *BCR::NTRK3* and *SPTAN1::NTRK2*. The *BCR::NTRK3* fusion retained the oligomerization domain of BCR together with the tyrosine kinase domain of NTRK3, a structure consistent with known oncogenic *NTRK* fusions and analogous to the *BCR::NTRK2* fusion described by Jones et al. [66]. The *SPTAN1::NTRK2* fusion joined exon 55 of *SPTAN1* with exon 14 of *NTRK2* (Figure 6); *SPTAN1* has previously been described as a fusion partner in T‐ALL with ABL1 [67]. Both novel fusions extend the spectrum of NTRK‐driven pLGG.

## 5 Conclusion

Our study underscores that the genetic landscape of pediatric LGG is dominated by alterations converging on the MAPK pathway, with *BRAF*, *FGFR*, *RAF1*, and *NTRK* events representing key diagnostic and therapeutic targets. Molecular stratification is no longer optional but essential, as effective inhibitors against *BRAF* p.V600E and MEK are already in clinical use, and agents directed at FGFR and other tyrosine kinases are rapidly emerging. Accurate detection of oncogenic fusions is therefore central to diagnosis, prognostication, and treatment selection. Implementing optimized diagnostic algorithms with robust multicaller strategies will be critical to reliably distinguish true driver events from benign fusions or technical artifacts, ultimately enabling precision medicine for all patients with LGG.

## Author Contributions

**Petr Brož:** writing – original draft, investigation. **Martina Strnadová:** methodology‐NGS, editing. **Denisa Olejníková:** methodology‐NGS, editing. **Johana Kotiš:** methodology‐NGS, editing. **Tereza Pospíšilíková:** methodology‐NGS, editing. **Adéla Mišove:** PCR, RT‐PCR, editing. **Miroslav Koblížek:** morphology, review and editing. **Josef Zámečník:** morphology, review and editing. **Michal Zápotocký:** clinical data, review and editing. **David Sumerauer:** clinical data, review and editing. **Aleš Vícha:** investigation, review and editing. **Martin Kynčl:** imaging processes, review and editing. **Petr Libý:** neurosurgery, editing. **Vladimír Beneš:** neurosurgery, editing. **Lenka Krsková:** writing – original draft, investigation, validation, supervision. All authors read and approved the final manuscript.

## Ethics Statement

The present study was approved by the ethics committee of the University Hospital Motol (reference no. EK‐97/25) and adhered to the tenets of the Declaration of Helsinki.

## Conflicts of Interest

The authors declare no conflicts of interest.

## Supporting information


**Table S1:** Primer table for verification of NGS results.

**Data S1:** Supporting Information.

## Data Availability

The data that support the findings of this study are available on request from the corresponding author. The data are not publicly available due to privacy or ethical restrictions.
