# Supplementary material for: Molecular Landscape of Pediatric Low‐Grade Gliomas: Insights From RNA‐NGS and Bioinformatic Analysis
Source: Genes Chromosomes Cancer. 2025 Oct 11;64(10):e70085. doi: 10.1002/gcc.70085 (PMC12514749; doi:10.1002/gcc.70085)
Supplement: Supplementary file 1 — Table S1: Primer table for verification of NGS results. [file GCC-64-e70085-s001.docx]

Supplementary Table – Primer Table for Verification of NGS Results

| Gene | Sequence (5´-3´) |
| --- | --- |
| KIAA1549 Exon 13 | GGGTCCCCAGTAAGATCCAG |
| BRAF Exon 11 | CCCACTGTAATCTGCCCATC |
| KIAA1549 Exon 10 | TGGTGGTGATGGTGATTGTT |
| BRAF Exon 9 | GGGGTAGCAGACAAACCTGT |
| CLIP2 | TGCTCAAGGCACAGCATGAG |
| NTRK2 | TCCCATTGGAGATGTGATGGA |
| QKI | GCAGCTGATGAACGACAAGA |
| RAF1 | AGAACCACTCCAGCGTGACT |
| KANK1 | GACACTGCTGGCTGAGAACTAC |
| NTRK2 | AGTCATCATCATTGCTGATAAC |
| BRAF | AGCAGATGAAGATCATCGAAATC |
| GNAI1 | TGGAGCGGAGTAAGATGATCGA |
| BCAS1 | TGGACACGAACTCACTGCAGAATG |
| BRAF | AGTGAGCCAGGTAATGAGGCAGGG |
| GTF21 | GTATGGAATCCCAAGGCTGG |
| BRAF | TCACGTTAGTTAGTGAGCCAG |
| MKRN1 | AGTGGGAGAGTGCCGATACG |
| BRAF | TCACTGCCACATCACCATGC |
| NUDCD3 | AGAAGAGCCCATCGACATTGA |
| BRAF | TTAGTGAGCCAGGTAATGAGGC |
| SPTAN1 | GAGAACGTCAAGTCCAGCGA |
| NTRK2 | GCCAAACTTGGAGTGTCTTGC |
| FGFR3 | CAACTGCACACACGACCTG |
| TACC3 | CCTCTTCGAACCTGTCCATGA |
| FGFR2 | TGTACATGATGATGAGGGACTGTT |
| PASD1 | AAGTCTTGCAAGGATAAGGCTG |
| KIAA1549 | GAATTGGAGCTCAGCCAGTGGAA |
| BRAF | CATCACCACGAAATCCTTGGTCTC |
| GOPC | TGTGGCTCCTGAAGTGGATTCTGA |
| ROS1 | CAGCTCAGCCAACTCTTTGTCTTC |
| TAX1BP1 | AACAGCACAACTTCGAGAACAAGT |
| BRAF | GTTAGTGAGCCAGGTAATGAGGCA |
| PPP1CB | CTGTTGTCATGGAGGATTGTC |
| ALK | GGTTGTAGTCGGTCATGATGGTCG |
| FGFR2 | CACAGAATGGATAAGCCAGCCTACT |
| KIAA1598 | AGGTCCTTCTCCAGCTCCAGGT |
| FGFR1 | TGATGCGGGACTGCTGGCATG |
| TACC1 | TCTGATTCGCTGAGGCAGATCC |
| EPHB2 | CAGACAAGCATCCAGGAGAAGT |
| BRAF | CATTCGATTCCTGTCTTCTGAG |
| NRF1 | CATGCTGTCGCCACCCTGGCTGA |
| BRAF | CTGAGGATGAAGATGACTTC |
| PAG1 | TGTGGGGAAGTCTGGCTGCTGT |
| BRAF | CTTGTAGACTGTTCCAAATGATC |
| PDE4DIP | CTGATGTCCCAAGCACCCATCTCT |
| NTRK1 | AGCTGCCACCCAATGTCATGAAAT |
| FGFR2 | CAGAATGGATAAGCCAGCCAACTG |
| INA | CTGTAAGCTGCTATCTCAATGTC |
| FGFR2 | GAATGGATAAGCCAGCCAACT |
| ZCCHC24 | TGAGGGTCAGGTCTGAGAAGTG |
| PRKAR2B | TCAACTTCGCCGAGGAGCCCATGC |
| BRAF | CATTCGATTCCTGTCTTCTGAG |
| FAM131B | ATGAGGTGATTGCAGTGGATTGGA |
| BRAF | GGGGTAGCAGACAAACCTGT |
| RNF130 | GGCTCTCTAGTCTTCGTGTCA |
| BRAF | GGGGTAGCAGACAAACCTGT |
| NACC2 | CCACCTTCTTTGACAGGAACACAC |
| NTRK2 | TCCGAAGAAGATGGAGTGTTACTC |
| KANK1 | GACACTGCTGGCTGAGAACTAC |
| NTRK2 | AGTCATCATCATTGCTGATAAC |
| KIF21B | GAGGATGAAGATGAGGACTCG |
| NTRK1 | CCAATGTCATGAAATGCAGGG |
| BCR | AGATCTGGCCCAACGATGGCGA |
| NTRK3 | AACTTGACAATGTGCTCATGC |
| GIGYF2 | CCAAGGAGTTTGCCAAGCAGTTC |
| ALK | GCACCTCCTTCAGGTCACTGAT |
| GIT2 | ACCAGACACAGCAGAACCCCA |
| ROS1 | CAGCAAGAGACGCAGAGTCAGT |
| SRGAP | ATGCCACCATGCAGACATTA |
| RAF1 E | CATTCCCTGAGCCGTCTG |
| FGFR1 Exon 18-KDD FGFR1 | CATGCCCCTGGACCAGTA |
| FGFR1 Exon 10-KDD FGFR1 | TCTTCGGGAAGCTCATACTCA |
