# Supplementary material for: Molecular Landscape of Pediatric Low‐Grade Gliomas: Insights From RNA‐NGS and Bioinformatic Analysis
Source: Genes Chromosomes Cancer. 2025 Oct 11;64(10):e70085. doi: 10.1002/gcc.70085 (PMC12514749; doi:10.1002/gcc.70085)
Supplement: Supplementary file 2 — Data S1: Supporting Information. [file GCC-64-e70085-s002.docx]

**Supplementary methods**

Bioinformatics Methodology

For the transcriptome analysis, we utilized the Gencode GTF file version 45 (GRCh38.p14) (1). Raw sequencing data were first processed with Fastp, a fast and efficient tool for quality control and preprocessing of high-throughput sequencing data) (2). To assess the global quality of the trimmed data, we used FastQC (3).

We employed a custom trimming setup that included the parameters --trim_poly_g, --trim_front, and --trim_tail, which target poly-G sequences and the removal of adapters from the 5’ and 3’ ends of the reads. This ensures a more accurate downstream analysis by reducing potential biases from poor-quality regions and adapter contamination. Fastp is particularly efficient in removing unwanted sequences without losing too much useful data (2).

To assess the global quality of the trimmed data, we used FastQC, an algorithm designed to provide an overview of the quality scores, GC content, and sequence length distribution, among other metrics, across the dataset. This initial quality assessment is crucial for identifying any potential issues in the sequencing process that could impact the reliability of the subsequent analyses (3).

References

1. Kilday JP, Bartels UK, Bouffet E (2014) Targeted therapy in pediatric low-grade glioma. Current Neurology and Neuroscience Reports 14(4):441. doi: 10.1007/s11910-014-0441-0.
2. Chen S, Zhou Y, Chen Y, Gu J (2018) fastp: an ultra-fast all-in-one FASTQ preprocessor. Bioinformatics 34(17):i884-i890. doi: 10.1093/bioinformatics/bty560.
3. Andrews, S. (2010) FastQC: A Quality Control Tool for High Throughput Sequence Data.
